# Ten years of progress in radiation oncology

**DOI:** 10.1186/1471-2407-11-503

**Published:** 2011-11-30

**Authors:** Dirk Vordermark

**Affiliations:** 1 Department of Radiation Oncology, Martin Luther University Halle-Wittenberg, Halle (Saale), Germany

## Abstract

Over the last decade, *BMC Cancer *has continuously published important research from the field of radiation oncology. Major developments in this field include the introduction of new imaging modalities into radiotherapy planning, the availability of hardware and software for more precise delivery of radiation dose, the individualization of radiotherapy concepts, for example, based on microarray data, and the combination of radiotherapy with molecular targeting approaches to overcome the radioresistance of tumor cells.

## Review

On the occasion of the 10^th ^anniversary of *BMC Cancer*, this mini-review will address major developments in the field of radiation oncology over the last decade. Important contributions published in this journal will be discussed.

Radiation oncology is a cornerstone of modern multidisciplinary cancer treatment. It has a place in the management of most common types of cancer, either as a single modality and organ-preserving alternative to surgery, for example, in organ-confined prostate cancer, or as an element in a sequence of treatment steps, such as in adjuvant radiotherapy after breast-conserving surgery for breast cancer.

From the launch of *BMC Cancer*, clinical and experimental contributions from radiation oncology and radiation biology have had a special place in this journal. The very first truly radiotherapy-related paper published in this journal on 19 June 2001, a meta-analysis by Meert *et al*. on the role of prophylactic cranial irradiation in small-cell lung cancer, was present on the most-viewed list of the journal for many years [1].

Strategies to improve the outcome of radiotherapy have aimed to improve tumor control rates, thereby increasing the chances of a cure in radical or adjuvant therapy or to increase the rates of symptom response in palliative situations. At the same time, reduction of toxicity and late effects was also intended, for example, by lowering of radiation dose to normal tissues adjacent to the tumor target volumes.

The availability and implementation of new technology as well as rigorous experimental, translational and clinical studies have advanced the field of radiation oncology in the last decade. Most progress was made in the following areas: imaging of tumor morphology and function for radiotherapy planning, precision of radiotherapy delivery, individualization of radiotherapy concepts and the modification of tumor cell radiosensitivity by molecular targeting.

### Imaging for radiotherapy planning

Computed tomography (CT) scans acquired in the radiotherapy treatment position before the start of radiotherapy remain the basic imaging modality for contouring tumor target volumes and healthy tissues ("organs at risk") as well as for dose calculation in radiotherapy planning. As dose-response relationships have been demonstrated for several tumor types ("higher dose to the tumor = better chance of cure"), for example, in radical radiotherapy of prostate cancer or non-small-cell lung cancer, efforts to increase the radiotherapy dose in limited tumor volumes with small margins were undertaken. However, the inability of CT to provide functional information, for example, on tumor vitality, proliferation, oxygenation or perfusion and the problem of day-to-day organ motion have necessitated additional information to advance radiotherapy planning.

Functional imaging modalities, such as magnetic resonance imaging spectroscopy (MRS), and, in particular, positron-emission tomography (PET) have opened new possibilities to obtain metabolic information and identify the most radioresistant subvolumes within a tumor [2]. MRS-defined dominant tumor lesions, for example, in the prostate, can be specifically addressed by an escalated radiotherapy dose [3].

### Precision of radiotherapy delivery

Extremely precise delivery of high radiation doses to small volumes was already technically possible in the 1990s and favorable results were obtained in benign and malignant brain tumors with a few fractions ("hypofractionated") or single-fraction stereotactic radiotherapy ("radiosurgery") [4]. The main indications for this technique are brain metastases, recurrent (previously irradiated) malignant gliomas, vestibular schwannomas and meningiomas. The brain is ideal for this procedure, as tumor or organ motion is practically non-existent.

**Figure 1:**
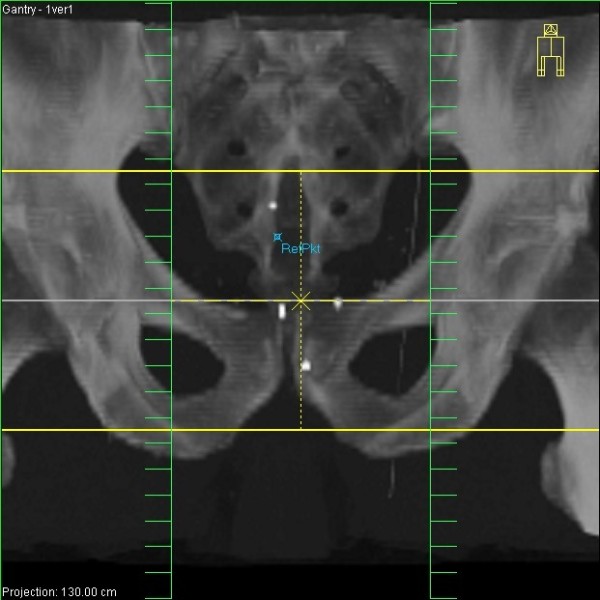
**Visualization of three gold markers implanted into the prostate on a reconstructed CT image**. The prostate itself is not visible, but the three intraprostatic markers can be used for daily image-guided radiotherapy (IGRT) with online adaptation of the beams to the current prostate position.

The problem of motion of tumor-bearing organs as well as adjacent healthy organs, most prominently exemplified by day-to-day motion of the prostate due to varying filling states of the rectum and of lung tumor movement within the breathing cycle, has been addressed by the implementation of image-guided radiotherapy (IGRT). Whereas only boney structures could be visualized in the past on the treatment couch of the linear accelerator at the time of each radiotherapy fraction, the integration of computed tomography into linear accelerator technology ("cone-beam CT") as well as the option to introduce radio-opaque fiducial markers into tumors or tumor-bearing organs, such as the prostate (Figure 1), made possible the correction of the patient position based on this information at each treatment session, thereby drastically reducing the margins around the tumor/organ required to compensate for motion.

**Figure 2:**
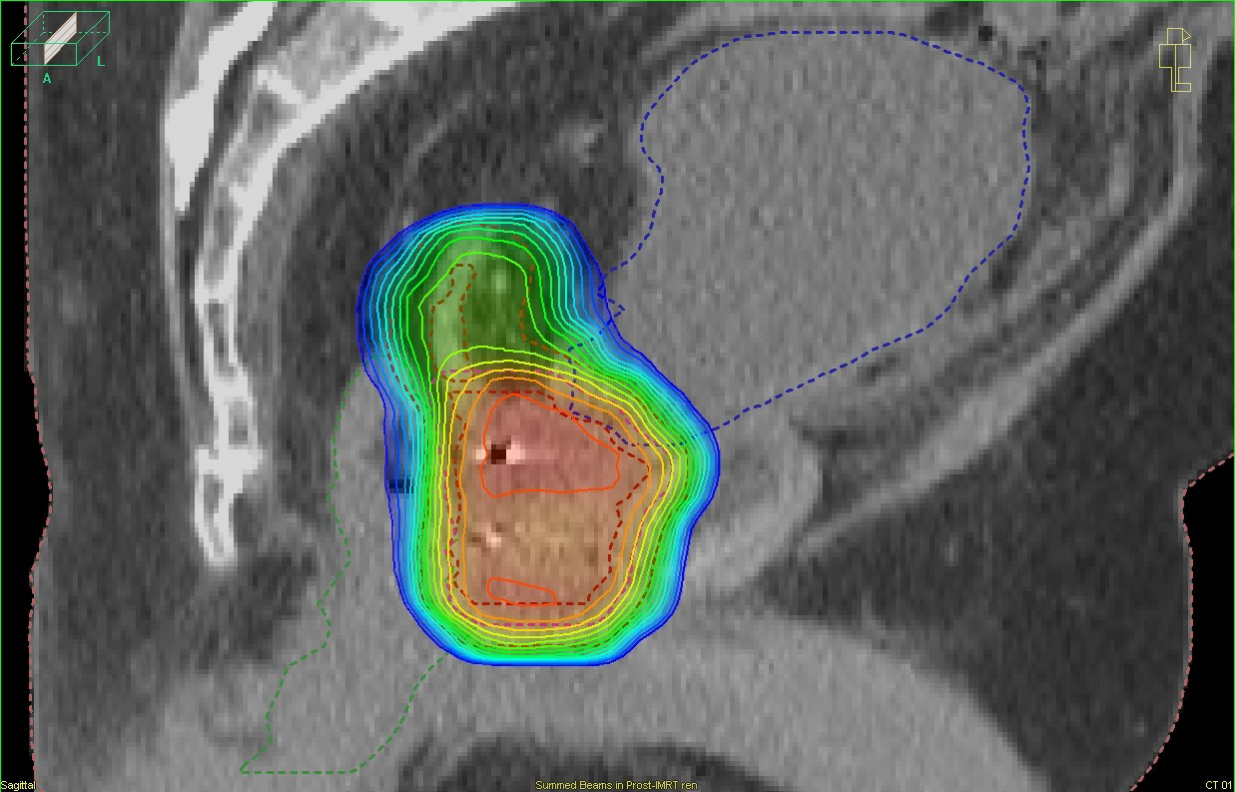
**Intensity-modulated radiotherapy (IMRT) dose distribution for prostate cancer in a sagittal CT reconstruction**.

Such advanced imaging on the treatment table was a prerequisite for the clinical introduction of advanced algorithms of dose calculation and delivery. Intensity-modulated radiotherapy (IMRT) enabled radiation physicists to create treatment plans with highly individualized dose distributions and a sharp dose gradient at the interface of tumor volume and healthy organ, even if the latter is virtually enclosed by the former [5]. Typical examples include the sparing of the highly radiosensitive parotid glands in radiotherapy of head and neck cancer and the protection of rectal mucosa adjacent to prostate and seminal vesicles (Figure 2). Sophisticated target volumes based on functional imaging data, IGRT and IMRT have been integrated in novel radiotherapy concepts [6]. Tomotherapy, an advanced type of IMRT, integrates imaging of the patient and delivery of radiotherapy in a sectional manner [7].

Proton radiotherapy, due to advantageous physical properties, has the potential to further improve clinical results so far achievable with modern linear accelerator photon radiotherapy. Like recent improvements in photon delivery, increased (biologically effective) doses in the tumor volume and/or reduced radiation dose in healthy organs - as achievable with protons according to theoretical planning studies - may further improve the therapeutic ratio of radiotherapy. However, clinical trial data is needed to fully assess the potential of proton radiotherapy [8].

### Individualiziation of radiotherapy concepts

In the past, based on the results of large randomized trials and meta-analyses, specific recommendations for the delivery of radiotherapy were made for tumor entities and disease stages. Even today, such statements in national and international guidelines for cancer treatment define the standards of care. However, the assessment of tumor material in individual patients has been proposed as a predominant source of information on which to base treatment decisions. Specific combinations of biomarkers detectable by immunohistochemistry (tissue microarrays) and specific gene signatures detectable in gene microarray studies have been used predominantly to predict the benefit from postoperative chemotherapy. While a focus of this field has been to identify subgroups of breast cancer patients benefiting from particular types of systemic therapy, response to radiotherapy has equally been addressed by microarray studies, for example, in diseases treated with radical radiotherapy such as cervix cancer [9].

### Molecular targeting

Experimental studies of radiosensitivity of tumor cells in *in-vitro *and *in-vivo *models have identified important mechanisms of radioresistance. Some of these findings could already be translated into clinically useful protocols of radiotherapy in combination with molecular targeting agents. The most prominent example is targeting of the epithelial growth factor receptor (EGFR) in combination with radiotherapy. Initially, the association of EGFR overexpression with prognosis was assessed in several tumor types [10]. In a randomized trial in head and neck cancer, EGFR targeting improved the outcome compared to radiotherapy alone, leading to further trials of treatment intensification with more complex drug combinations as well as to new translational research initiatives [11].

Low tumor oxygenation is a frequently observed cause of poor response to radiotherapy, for example, in head and neck or cervix cancer. Normalizing tumor oxygenation and specifically targeting or radiosensitizing hypoxic tumor cells have been alternative strategies to improve the tumor control rates in hypoxic tumors. Recently, hypoxia-related molecules have been assessed as targets in combination with radiotherapy, showing some potential for radiosensitization of tumor cells [12].

## Conclusions

Ten years of *BMC Cancer *have accompanied a decade of rapid development in the field of radiation oncology and its technical, clinical, biological and translational research branches. While this decade has also seen dramatic changes in the area of open-access publishing, *BMC Cancer *continues to be a platform for radiotherapy-related contributions in an interdisciplinary oncology setting.

## Abbreviations

CT: computed tomography; EGFR: epithelial growth factor receptor; IGRT: image-guided radiotherapy; IMRT: Intensity-modulated radiotherapy; MRS: magnetic resonance imaging spectroscopy; PET: positron-emission tomography.

## Competing interests

The author declares that they have no competing interests.

## Authors' contributions

DV individually drafted and revised this manuscript.

## Pre-publication history

The pre-publication history for this paper can be accessed here:

http://www.biomedcentral.com/1471-2407/11/503/prepub
